# Cinobufagin Induces Cell Cycle Arrest at the G2/M Phase and Promotes Apoptosis in Malignant Melanoma Cells

**DOI:** 10.3389/fonc.2019.00853

**Published:** 2019-09-04

**Authors:** Zhaohai Pan, Xin Zhang, Pengfei Yu, Xiaoyu Chen, Peng Lu, Minjing Li, Xiaona Liu, Zhipeng Li, Fei Wei, Kejun Wang, Qiusheng Zheng, Defang Li

**Affiliations:** ^1^Yantai Key Laboratory of Pharmacology of Traditional Chinese Medicine in Tumor Metabolism, School of Integrated Traditional Chinese and Western Medicine, Binzhou Medical University, Yantai, China; ^2^School of Pharmacy, Binzhou Medical University, Yantai, China; ^3^Yantai Affiliated Hospital of Binzhou Medical University, Yantai, China; ^4^School of Public Health and Management, Binzhou Medical University, Yantai, China; ^5^Key Laboratory of Xinjiang Endemic Phytomedicine Resources of Ministry of Education, School of Pharmacy, Shihezi University, Shihezi, China

**Keywords:** cinobufagin, melanoma, A375 cell, mitochondria-mediated apoptosis, cell cycle arrest, G2/M phase

## Abstract

Emerging evidence has shown that cinobufagin, as an active ingredient of Venenum Bufonis, inhibits tumor development. The aim of this study was to investigate the inhibitory effects of cinobufagin on A375 human malignant melanoma cells. MTT and colony formation assays showed that cinobufagin significantly inhibited A375 cell proliferation and cell colony formation. Additional studies demonstrated that cinobufagin markedly increased the levels of ATM serine/threonine kinase (ATM) and checkpoint kinase 2 (Chk2) and decreased the levels of cell division cycle 25C (CDC25C), cyclin-dependent kinase 1 (CDK1), and cyclin B, subsequently inducing G2/M cell cycle arrest in A375 cells. Moreover, cinobufagin clearly inhibited the levels of phosphoinositide 3-kinase (PI3K), phosphorylated PI3K (p-PI3K), AKT, p-AKT, and B-cell lymphoma 2 (Bcl-2). By contrast, it increased the levels of Bcl-2-associated death promoter, Bcl-2-associated X, cytoplasmic cytochrome C, and apoptotic protease activating factor 1, leading to increased levels of cleaved caspase-9 and cleaved caspase-3, resulting in the apoptosis of A375 cells. Together, these results indicate that cinobufagin can induce cell cycle arrest at the G2/M phase and apoptosis, leading to inhibition of A375/B16 cell proliferation. Thus, cinobufagin may be useful for melanoma treatment.

## Introduction

Malignant melanoma is the deadliest type of skin cancer. It can develop from benign borderline nevus or mixed nevus and, once formed, is highly invasive and metastatic (1–3). The etiology of malignant melanoma has not been fully clarified (4). The current mainstream view is that its occurrence is mainly and strongly associated with solar radiation, ultraviolet radiation, ionizing radiation, and gene mutation (5). Ultraviolet radiation is the main external factor causing malignant melanoma (6), as reports have shown that intense ultraviolet rays destroy proteins in skin cells, leading to this disease (7). In addition, race and ethnicity are also important factors in the development of malignant melanoma. Compared with Asians and people of African descent, Caucasians are more likely to develop malignant melanoma (8–10).

At present, surgical resection is the main treatment for early non-metastatic melanoma, and the cure rate can reach up to more than 90% (11), whereas radiotherapy and chemotherapy are the main treatments for malignant melanoma (12). Currently, 5-fluorouracil (5-FU), cisplatin, and alkylating agents with cytostatic activity are commonly used for the treatment of malignant melanoma (13), of which the main role of the chemotherapy agents is to cause irreversible damage to DNA in tumor cells. These types of drugs can cause tumor cells to stagnate in a specific cell cycle and fail to divide and proliferate (14). At present, inhibiting the metastasis and spread of melanoma is one of the goals of early treatment. However, once melanoma has spread and metastasized, treatment is extremely difficult (15). There is currently no effective treatment for metastatic melanoma; thus, the identification of novel drugs for the treatment of this disease is urgently needed.

Cinobufagin is one of the main active components extracted from the Traditional Chinese Medicine Venenum Bufonis (16). Initially, cinobufagin as a painkiller was primitively used to treat pain caused by cancer such as liver, prostate, and breast cancers (16). Recently, it has been reported that cinobufagin can effectively inhibit the proliferation of tumor cells by inducing apoptosis and cell cycle arrest in several tumor cells (e.g., hepatocellular carcinoma Huh-7 cells, colorectal cancer HCT-116 and breast cancer MCF-7 cells) (17–21). Moreover, cinobufagin is one of the chemotherapeutic drugs approved by Chinese State Food and Drug Administration for the treatment of liver and prostate cancers in China (22). However, the effects of cinobufagin on malignant melanoma have not been studied. So, we employed melanoma A375 cells and B16 cells to explore the anti-tumor effects of cinobufagin.

In this study, the anti-tumor effects and molecular mechanisms of cinobufagin in A375 malignant melanoma cells *in vitro* was studied for the first time. The results showed that cinobufagin arrested A375 cells at the G2/M phase of the cell cycle and effectively induced apoptosis. Thus, cinobufagin may be a potential drug for the treatment of malignant melanoma.

## Materials and Methods

### Cell Culture

Human malignant melanoma A375 cells (Cat no. SCSP-533) and mouse melanoma B16 cells (Cat no. TCM-2) were ordered from the Cell Bank, Typical Culture Preservation Commission, Chinese Academy of Sciences (Shanghai, China). Cells were cultured with Dulbecco's Modified Eagle's Medium (DMEM)/High glucose (Cat no. SH30243.01B; Hyclone, Logan, UT, USA) containing 10% fetal bovine serum (Cat no. 10091148; Gibco, Invitrogen, Shanghai, China), 1% sodium pyruvate (Cat no. SP0100; Solarbio, Beijing, China), 0.1 U/L penicillin, and 0.1 μg/L streptomycin (Cat no. P1400; Solarbio, Beijing, China). The cells were incubated in 5% CO_2_ incubator (HF90, Heal Force Bio-meditech Holdings Limited, Shanghai, China) at 37°C for 48 h and then propagated.

### MTT Assay

The viability of A375/B16 cells after treatment with different concentrations of cinobufagin (Purity: 98%; Cat no. 237113; J&K Scientific Ltd., Beijing, China) was detected by the MTT assay (23). Adherent A375/B16 cells in logarithmic growth period were digested with trypsin-EDTA solution (Cat no. T1320; Solarbio, Beijing, China), and then re-suspended into 1 × 10^5^/mL cell suspensions. The cell suspension was inoculated into 96-well plates with 100 μL per well. After incubation for 24 h, the cells were treated with different concentrations of cinobufagin for 24 and 48 h. Then 10 μL MTT solution (5 mg/mL) (Cat no. M1020, Solarbio Life Sciences, Beijing, China) was added to each well and incubated for 2 h. Next, the culture medium was discarded, 150 μL dimethyl sulfoxide (DMSO) was added to each well to dissolve the formazan crystals, and the absorbance of each well was measured at 490 nm (24). Cells treated with 0.1% DMSO in DMEM were used as the control group; the cell viability of this group was 100%. The IC_50_ represents the concentration of cinobufagin that reduced cell viability to 50%.

### Colony Formation Assay

A375 cells were digested and plated in 6-well plates at a density of 300 cells per well. After incubating in a constant temperature incubator for 24 h, different concentrations of cinobufagin were added to the cells and cultured for 24 h. Then the medium containing the drug was discarded and replaced with fresh culture. The culture medium was changed every 3 days for 14 days. Giemsa staining solution (Cat no. G1010, Solarbio, Beijing, China) was used to stain the cells, which were observed and photographed under an inverted microscope (DMI3000B; Leica Microsystems, Wetzlar, Germany). Colonies with more than 50 cells were counted to calculate the colony formation rate.

### Hoechst 33258 Staining

A375/B16 cells were inoculated on sterile cover glasses, cultured in a 6-well plate for 24 h, and treated with different concentrations of cinobufagin. After 24 h of treatment, cells on the cover glass were fixed and washed twice with phosphate-buffered saline (PBS). Then the cells were stained with Hoechst 33258 staining solution (Cat no. C1018; Beyotime, Shanghai, China) in the dark for 5 min. Finally, the cover glasses were attached to the slides and observed and photographed under a fluorescence microscope (DMI3000B; Leica Microsystems).

### Cell Cycle Analysis

A375/B16 cells were treated with different concentrations of cinobufagin for 24 h, and then collected by digestion and made into cell suspensions. The cells were fixed in pre-cooled 70% ethanol solution at 4°C for 2 h and then centrifuged at 800× g in a low temperature centrifuge for 5 min. The ethanol solution was discarded and the cells were washed twice with PBS. Then they were precipitated and suspended in 500 μL phosphate buffer containing 0.02 mg/mL propidium iodide (PI) (Cat no. ST512; Beyotime, Shanghai, China) and 0.1 mg/mL Ribonuclease A (Cat no. ST576; Beyotime, Shanghai, China). After 30 min incubation in the dark at 37°C, the cells were precipitated and suspended in phosphate buffer solution. The Epics XL Flow Cytometer (Beckman Coulter, Inc., Brea, CA, USA) was used to detect fluorescence of the PI-DNA complex, and Winmdi 2.8 software (Scripps Research, La Jolla, CA, USA) was used to analyze the cell distribution at different stages of the cell cycle (25).

### Annexin V-FITC/PI Double Staining Assay

A375/B16 cells were treated with different concentrations of cinobufagin for 24 h, after which they were digested and made into cell suspensions (8 × 10^5^ cells were collected from each concentration group). Cells were centrifuged at 800× g, and then re-suspended with a pre-configured mix of dye consisting of 195 μL Annexin V-FITC binding buffer, 10 μL PI solution, and 5 μL Annexin V-FITC solution (Annexin V-FITC Apoptosis Assay Kit, Cat no. C1062L; Beyotime, Shanghai, China). The new cell suspension was placed in the dark for 15 min. The reaction was terminated by adding 400 μL 1X binding buffer. The FACSCanto II flow cytometer (Becton Dickinson, Franklin Lakes, NJ, USA) was used to detect the intensities of red and green fluorescence, and the apoptotic rates of cinobufagin-treated cells were analyzed using FACSDiva software (version 6.1.3; Becton Dickinson).

### Western Blot Analysis

A375 cells (1 × 10^6^ cells) were inoculated in 10-cm culture dishes. After incubation for 12 h, different concentrations of cinobufagin were added for treatment. After 24 h, trypsin without EDTA was used for digestion, followed by centrifugation, and the addition of RIPA lysis solution (Cat no. P0013B; Beyotime, Shanghai, China) to the cell pellet containing 150 mM NaCl, 50 mM Tris, 1 mM EDTA, 1% Triton X-100, 1% sodium deoxycholate, 2.5 mM sodium pyrophosphate, 0.1 mM sodium orthovanadate, 0.5 mM dithiothreitol, 0.1 mM phenylmethanesulfonylfluoride, and 1X protease inhibitor (the pH was adjusted to 7.4). The cells were incubated for 30 min at −4°C and placed on a vortex oscillator for 30 s every 10 min. The supernatant was collected and stored at −20°C. Protein concentrations were determined using the BCA Protein Assay Kit (Cat no. P0010; Beyotime, Shanghai, China). Cell lysates with a protein content of 40 mg were mixed with an equal volume of sodium dodecyl sulfate (SDS) loading dye (2% SDS, 10% sucrose, 0.002% bromophenol blue, 5% 2-mercaptoethanol, 625 mM Tris; pH 6.8), and subsequently separated on 12.5% SDS-PAGE gels along with a rainbow-colored protein molecular marker (Cat no. PR1920, Solarbio, Beijing, China) for 2 h at 110 V. Then the proteins were electrophoretically transferred to polyvinylidene fluoride membranes (Millipore) using the Trans-Blot SD Semi-Dry Transfer Cell (Bio-Rad, Hercules, CA, USA). The membranes were blocked in 5% bovine serum albumin (BSA) in Tris-buffered saline containing 0.1% Tween-20 (TBST) for 60 min before being probed overnight at 4°C with the following primary antibodies (all from Abcam, Cambridge, UK): protein kinase B (AKT, 1:500, ab8805), phosphorylated AKT (p-AKT, 1:500, ab38449), phosphoinositide 3-kinase (PI3K, 1:1,000, ab140307), p-PI3K (1:1,000, ab38449), ATM serine/threonine kinase (ATM, 1:1,000, ab78), checkpoint kinase 2 (Chk2, 1:1,000, ab47433), p-Chk2 (1:1,000, ab3501), cell division cycle 25C (CDC25C, 1:1,000, ab32444), p-CDC25C (1:500, ab47322), cyclin-dependent kinase 1 (CDK1, 1:1,000, ab18), cyclin B (1:1,000, ab181593), Bcl-xL/Bcl-2-associated death promoter (BAD, 1:1,000, ab90435), Bcl-2-associated X (BAX, 1:1,000, ab32503), B-cell lymphoma-2 (Bcl-2, 1:1,000, ab32124), cytochrome C (1:1,000, ab13575), apoptotic protease activating factor-1 (Apaf-1, 1:1,000, ab2000), active caspase-3 (cleaved caspase-3, 1:1,000, ab32042), caspase-3, 1:1,000, ab13585), active caspase-9 (cleaved caspase-9, 1:1,000, ab2324), and caspase-9 (1:1,000, ab202068). Subsequently, the membranes were washed with TBST buffer for 45 min and then probed with appropriate horseradish peroxidase-conjugated secondary antibodies for 1 h. Immunoreactive bands were visualized by the Novex™ ECL Chemiluminescent Substrate Reagent Kit (WP20005; Thermo Fisher Scientific, Shanghai, China) using Film processor (BioSpectrum Imaging System, Upland, CA, USA), and the gray-scale values of each band were calculated by Image-Pro Plus 6.0 (IPP6) software (26).

### Statistical Analysis

The experiments were conducted at least three times. All the data are shown as the mean ± standard deviation. The Student's *t-*test, one-way analysis of variance (ANOVA), or two-way ANOVA were employed to analyze the statistical differences. The analyses were performed using SPSS 21.0 software package (version 21.0, SPSS Inc., Chicago, IL, USA). *P* < 0.05 were considered statistically significant.

## Results

### *In vitro* Anti-tumor Effects of Cinobufagin in A375 Cells

**Figure 1:**
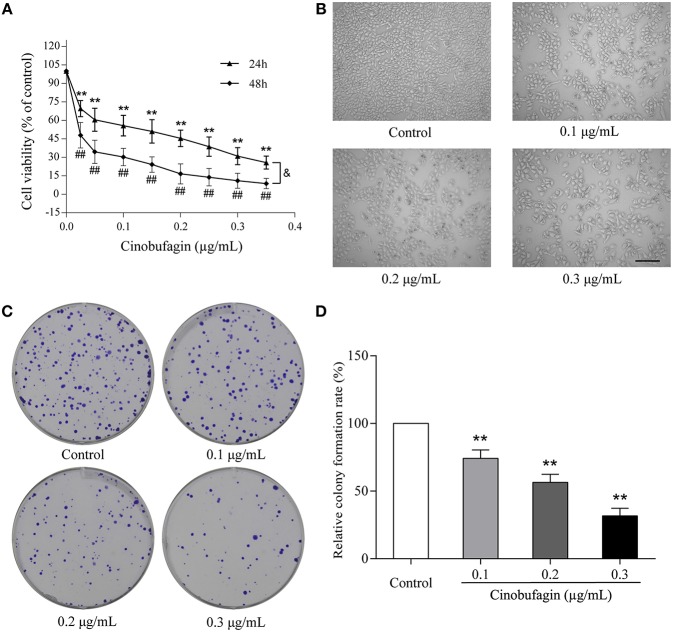
Inhibitory effects of cinobufagin on the proliferation of A375 cells. **(A)** A375 cells were inoculated into 96-well plates and treated with different concentrations of cinobufagin for 24 and 48 h, respectively; the cell viability was determined by the MTT assay. ^**^P < 0.01 vs. A375 cells control group (24 h); ^#^*P* < 0.05, ^##^*P* < 0.01 vs. A375 cells control group (48 h); ^&^*P* < 0.05 vs. A375 cells treated with different concentrations of cinobufagin for 24 or 48 h. **(B)** The morphologic changes of A375 cells treated with cinobufagin were observed by a phase contrast microscope. scale bar, 50 μm. **(C)** Representative images of the cell colony in cinobufagin-treated A375 cells. **(D)** Stastatical analysis of cell colony formation rate in cinobufagin-treated A375 cells. ^**^P < 0.01 compared with the control group. All data are presented as the mean ± SD from three independent experiments.

To investigate the effects of cinobufagin on malignant melanoma *in vitro*, A375 cells were treated with different concentrations of cinobufagin for 24 and 48 h, respectively. Then the activity of cinobufagin-treated A375 cells was detected by the MTT assay. The results showed that the proliferation of A375 cells was significantly inhibited after cinobufagin treatment for 24 h with an IC_50_ value of 0.2 μg/mL (Figure 1A). Treatment for 48 h with the same concentration of cinobufagin led to significantly higher proliferation than treatment with 24 h (Figure 1A). To confirm these inhibitory effects, the cell morphology was observed under a microscope after cinobufagin treatment. The number of A375 cells in the cinobufagin-treated groups was significantly less than that in the control group, and the cells showed varying degrees of deformation and shrinkage, some even falling off the surface of the petri dish (Figure 1B). In addition, cinobufagin effectively inhibited the colony formation of A375 cells compared with the control group (Figures 1C,D). These data showed that cinobufagin effectively inhibited A375 cell proliferation.

### Cinobufagin Induces Cell Cycle Arrest at the G2/M Phase in A375 Cells

**Figure 2:**
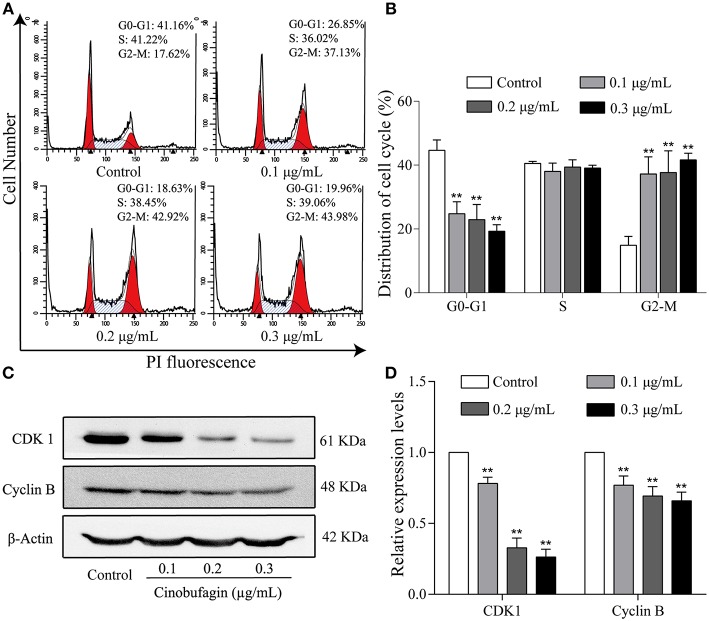
Cinobufagin induces cell cycle arrest at the G2/M phase in A375 cells. **(A)** Flow cytometry was used to determine the cell cycle distribution of A375 cells treated with cinobufagin. **(B)** Statistical analysis of the cell cycle distribution of A375 cells after cinobufagin treatment. **(C)** Levels of CDK1 and cyclin B proteins in cinobufagin-treated A375 cells were detected by western blotting. **(D)** Statistical analysis of relative protein levels of CDK1 and cyclin B in cinobufagin-treated A375 cells. ^**^P < 0.01 compared with the control group. All data are presented as the mean ± SD from three independent experiments.

To further investigate the inhibitory effects of cinobufagin on the proliferation of A375 cells, we examined the cell cycle distribution after a 24 h treatment with cinobufagin. The percentages of cinobufagin-treated A375 cells in the G2/M phase were significantly higher than those in the control group (Figures 2A,B). To verify this change, the levels of G2/M phase regulatory proteins (CDK1 and cyclin B) were examined by western blotting. The data showed that the levels of CDK1 and cyclin B were significantly decreased compared with the control group (Figures 2C,D). These results suggested that cinobufagin effectively induced G2/M phase cell cycle arrest by inhibiting the expression of CDK1 and cyclin B.

### Effects of Cinobufagin on the ATM/Chk2/CDC25C Signaling Pathway

**Figure 3:**
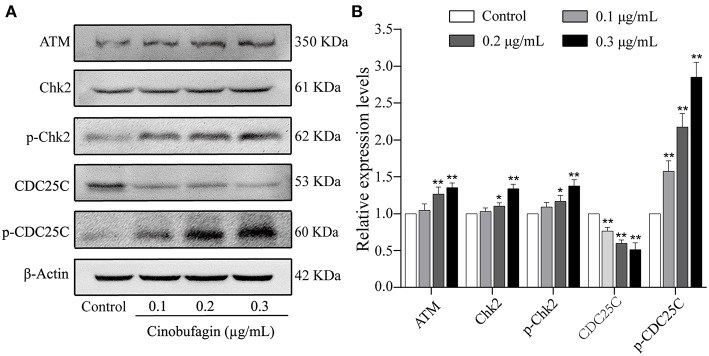
Effects of cinobufagin on the levels of ATM/Chk2/CDC25C signaling molecules. **(A)** The levels of ATM, Chk2, p-Chk2, CDC25C, and p-CDC25C in A375 cells treated with cinobufagin were detected by western blotting. **(B)** The relative protein levels of ATM, Chk2, p-Chk2, CDC25C, and p-CDC25C in cinobufagin-treated A375 cells were analyzed. The ratio of relative protein was standardized according to the protein levels in the control group. ^*^P < 0.05, ^**^P < 0.01 compared with the control group. All data are presented as the mean ± SD from three independent experiments.

The aforementioned results showed that cinobufagin could decrease the expression of CDK 1/cyclin B and induce cell cycle arrest at the G2/M phase in A375 cells. Considering that the ATM/Chk2/CDC25C signaling pathway plays an important role in regulating this phase of the cell cycle, we examined the levels of these proteins in cinobufagin-treated A375 cells. Western blot analysis showed that the protein levels of ATM, Chk2, p-Chk2, and p-CDC25C in cinobufagin-treated A375 cells were significantly higher, whereas the level of CDC25C was significantly lower than the control group (Figures 3A,B).

### Cinobufagin Promotes the Apoptosis of A375 Cells

**Figure 4:**
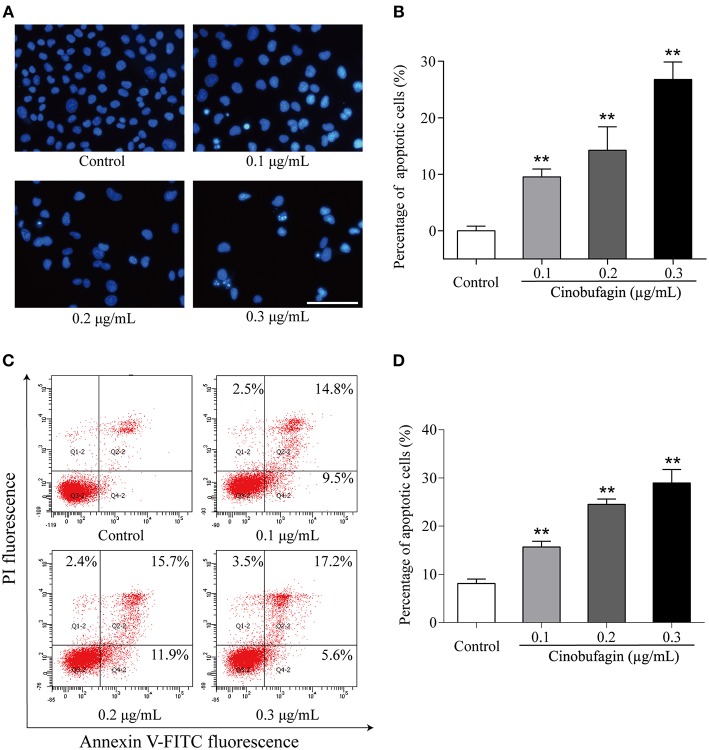
Cinobufagin induces A375 cell apoptosis. **(A)** Morphologic changes of cinobufagin-treated A375 cells were observed after Hoechst 33258 staining. **(B)** Percentages of apoptotic cells in cinobufagin-treated A375 cells were analyzed. **(C)** Annexin V-FITC/PI double staining was used to detect the apoptotic rate of A375 cells after cinobufagin treatment. **(D)** Statistical analysis of the apoptotic rate of A375 cell population after cinobufagin treatment. ^**^P < 0.01 compared with the control group. All data are presented as the mean ± SD from three independent experiments.

Based on the morphological changes of cinobufagin-treated A375 cells, cell shrinkage was observed under a light microscope (Figure 1B), indicating that cinobufagin might induce A375 cell apoptosis. Subsequently, Hoechst 33258 staining was used to determine if cinobufagin induced A375 cell apoptosis. The obvious apoptotic features including nuclear shrinkage, irregular condensation of chromatin, and apoptotic bodies were observed under a fluorescence microscope (Figures 4A,B). In addition, we used the Annexin V FITC/PI Double Staining Kit to measure the apoptotic rate of cinobufagin-treated A375 cells. Flow cytometry analysis showed that the percentages of apoptotic A375 cells significantly increased with the increase of cinobufagin concentration (Figures 4C,D). These results showed that cinobufagin effectively induced A375 cell apoptosis.

### Effects of Cinobufagin on Apoptosis-Related Proteins in A375 Cells

**Figure 5:**
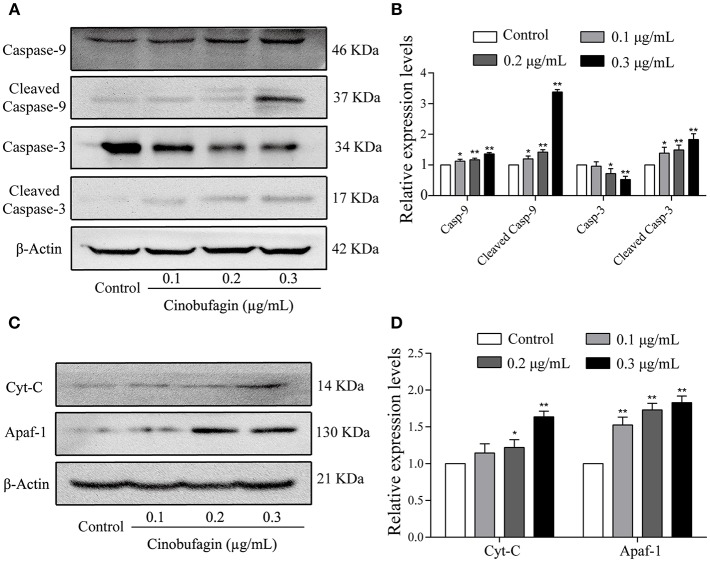
Effects of cinobufagin on the levels of apoptosis-related molecules. **(A)** Levels of caspase-9, cleaved caspase-9, caspase-3, and cleaved caspase-3 in cinobufagin-treated A375 cells were detected by western blotting. **(B)** The relative levels of caspase-9, cleaved caspase-9, caspase-3, and cleaved caspase-3 were analyzed. **(C)** The levels of Apaf-1 and cytoplasmic cytochrome C in cinobufagin-treated A375 cells were detected by western blotting. **(D)** The relative levels of Apaf-1 and cytoplasmic cytochrome C were analyzed. ^*^P < 0.05, ^**^P < 0.01 compared with the control group. All data are presented as the mean ± SD from three independent experiments.

In the aforementioned experiments, we observed that cinobufagin effectively induced the apoptosis of A375 cells. Thus, we next used western blotting to explore the changes of cell apoptotic molecules. Compared with the control group, the levels of cleaved caspase-3 and caspase-9 were significantly increased in cinobufagin-treated A375 cells, indicating that cinobufagin could simultaneous activate these proteins (Figures 5A,B). Furthermore, the upstream molecules (Apaf-1 and cytochrome C) levels of caspase-9 were examined by western blotting. The data showed that the levels of Apaf-1 and cytoplasmic cytochrome C were markedly higher than those in the control group (Figures 5C,D). These data showed that cinobufagin induced A375 cell apoptosis via a mitochondria-mediated apoptotic signaling pathway.

### Effects of Cinobufagin on the Upstream Molecules of Mitochondrial Apoptotic Signaling Molecules

**Figure 6:**
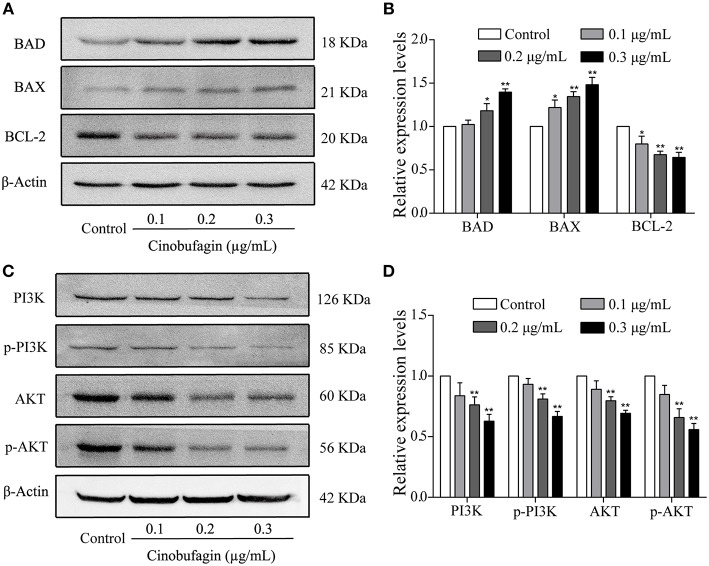
The regulatory effects of cinobufagin on the upstream molecules of mitochondrial apoptotic signaling molecules. **(A)** The levels of BAD, BAX, and Bcl-2 in cinobufagin-treated A375 cells were detected by western blotting. **(B)** The relative levels of BAD, BAX, and Bcl-2 were analyzed. **(D)** The levels of PI3K, p-PI3K, AKT, and p-AKT in cinobufagin-treated A375 cells were detected by western blotting. **(D)** Statistical analysis of the relative levels of PI3K, p-PI3K, AKT, and p-AKT in cinobufagin-treated A375 cells. ^*^P < 0.05, ^**^P < 0.01 compared with the control group. All data are presented as the mean ± SD from three independent experiments.

To verify that A375 cell apoptosis induced by cinobufagin was related to activation of the mitochondrial apoptotic pathway, we measured the levels of BAD, BAX, and Bcl-2 by western blotting. We found that the levels of pro-apoptotic BAD and BAX proteins were significantly increased and the level of anti-apoptotic Bcl-2 protein was markedly decreased in cinobufagin-treated A375 cells compared with control cells (Figures 6A,B). In addition, the protein levels of PI3K, p-PI3K, AKT, and p-AKT were clearly decreased in A375 cells after cinobufagin treatment (Figures 6C,D).

### Cinobufagin Induces Cell Cycle Arrest at the G2/M Phase and Cell Apoptosis in B16 Cells

**Figure 7:**
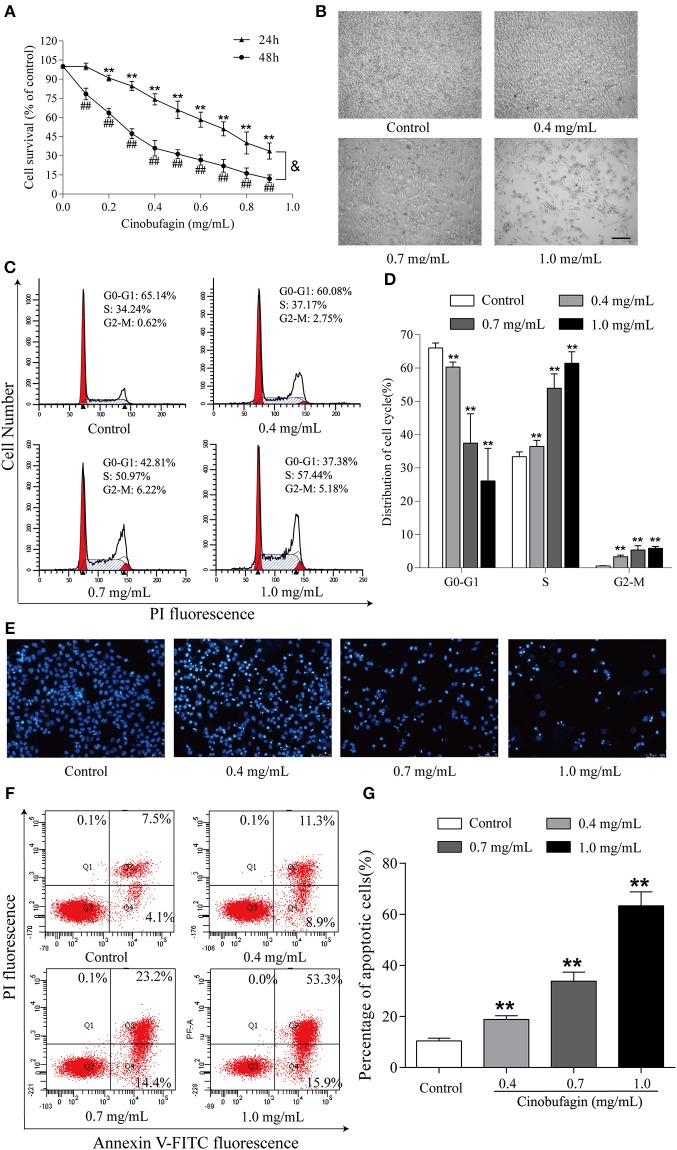
Cinobufagin induces cell cycle arrest and cell apoptosis in B16 cells. **(A)** The cell viability of cinobufagin-treated B16 cells was determined by the MTT assay. ^**^P < 0.01 vs. B16 cells control group (24 h); ^##^*P* < 0.01 vs. B16 cells control group (48 h); ^&^*P* < 0.05 vs. B16 cells treated with different concentrations of cinobufagin for 24 or 48 h. **(B)** The morphologic changes of B16 cells treated with cinobufagin were observed by a phase contrast microscope. scale bar, 50 μm. **(C)** Flow cytometry was used to determine the cell cycle distribution of B16 cells treated with cinobufagin. **(D)** Statistical analysis of the cell cycle distribution of B16 cells after cinobufagin treatment. **(E)** Morphologic changes of cinobufagin-treated B16 cells were observed after Hoechst 33258 staining. **(F)** Annexin V-FITC/PI double staining was used to detect the apoptotic rate of B16 cells after cinobufagin treatment. **(G)** Statistical analysis of the apoptotic rate of B16 cell population after cinobufagin treatment. ^**^P < 0.01 compared with the control group. All data are presented as the mean ± SD from three independent experiments.

Finally, we further explored the anti-tumor effects of cinobufagin in mouse melanoma B16 cells. Consistent with the data from human melanoma A375 cells, we found that the activity of B16 cells was also significantly inhibited after cinobufagin treatment in a dose- and time-dependent manner (Figure 7A). The number of B16 cells in the cinobufagin-treated groups was obviously less than that in the control group, and the cells showed varying degrees of deformation and shrinkage, some even falling off the surface of the petri dish (Figure 7B). Moreover, the percentages of cinobufagin-treated B16 cells in the G2/M phase were significantly higher than those in the control group (Figures 7C,D). Furthermore, Hoechst 33258 staining assay showed some obvious apoptotic features (e.g., nuclear shrinkage, irregular condensation of chromatin, and apoptotic bodies) in cinobufagin-treated B16 cells (Figure 7E). Flow cytometry analysis showed that the percentages of apoptotic cells in cinobufagin-treated B16 cells were significantly higher than that in the control group (Figures 4F,G). These results showed that cinobufagin effectively induced cell cycle arrest and cell apoptosis in B16 cells.

## Discussion

Venenum Bufonis is a traditional Chinese medicine that has been used in China for hundreds of years (20, 27). Previously, Venenum Bufonis and Venenum Bufonis extracts were mainly used as a cardiac stimulant and tumor analgesic (28). In recent years, a large number of studies have shown that the extract of Venenum Bufonis can inhibit the proliferation of some tumor cells (29). As one of the active components of Venenum Bufonis, cinobufagin has also entered garnered interest from researchers, as it has been reported that cinobufagin can effectively inhibit the proliferation of lung cancer cells (30), liver cancer cells (17), prostate cancer cells (31), and osteosarcoma cells (32) *in vitro*. In this study, we investigated the effects of cinobufagin on the proliferation of A375 human malignant melanoma cells and its intrinsic mechanism. The results showed that cinobufagin arrested A375 cells at the G2/M phase and induced apoptosis by activating mitochondrial apoptosis pathway.

Checkpoint is an important regulatory node of the cell cycle (1, 14, 33). Only through the checkpoint test can the cell enter the next cell cycle (34). The CDK1/cyclin B complex is a key regulatory factor of checkpoint in the G2/M phase (35). The complex is synthesized in large quantities when the cell passes through the G2/M phase (36). The content of CDK 1/cyclin B complex decreased correspondingly when G2/M cycle arrest occurred (37). In our study, we found that the levels of CDK1 and cyclin B in A375 cells were decreased after cinobufagin treatment. This result suggests that cinobufagin can decrease the levels of CDK1 and cyclin B, thereby decreasing the content of the CDK1/cyclin B complex, leading to A375 cell arrest at the G2/M phase.

Once cell cycle arrest occurs in the course of cell division, it is suggested that damage and error are difficult to repair during cell division (38). Cell cycle arrest at the G2/M phase indicates that the damage of intracellular DNA is difficult to repair (39). It has been reported that the ATM/ATR signaling pathway is activated when intracellular DNA is damaged (40), and can repair damaged DNA by regulating the activity of many proteins (41). It is widely believed that the ATM protein is activated during DNA damage, which subsequently upregulates the expression of Chk2 protein and increases the phosphorylation of Chk2 and CDC25C, leading to inhibition of the formation CDK1/cyclin B complex and cell cycle arrest at the G2/M phase (42, 43). In our study, we found that cinobufagin increased the levels of ATM and Chk2 and the phosphorylation level of Chk2 and CDC25C in A375 cells. Meanwhile, cinobufagin decreased the content of the CDK 1/cyclin B complex by inhibiting the levels of CDK1 and cyclin B, and subsequently inducing cell cycle arrest at the G2/M phase. Therefore, cinobufagin may induce DNA damage and ATM/ATR signaling pathway activation, leading to A375 cell arrest at the G2/M phase.

Mitochondria-mediated caspase cascade activation, as an important apoptotic signaling pathway, can be initiated by many stimuli (44). When exogenous stimuli activate BAX and Bcl-2 in the mitochondria, the membrane permeability of mitochondria changes (45). Subsequently, cytochrome C from mitochondria is released into the cytoplasm in large quantities (46). Cytochrome C in the cytoplasm activates Apaf-1, caspase-9, and caspase-3, leading to apoptosis (47). A previous study showed that cinobufagin induces human prostate cancer cell (LNCaP and DU145 cells) apoptosis via the mitochondria-mediated apoptotic signaling pathway (31). Consistent with the abovementioned studies, we found that cinobufagin induced A375 cell apoptosis after 24 h of treatment. Furthermore, the level of Bcl-2 was clearly decreased, whereas the levels of cleaved caspase-3/9, Apaf-1, cytoplasmic cytochrome C, BAD, and Bax were significantly increased in cinobufagin-treated A375 cells. These studies demonstrated that cinobufagin could induce A375 cell apoptosis through the mitochondria-mediated apoptotic signaling pathway.

The PI3K/AKT signaling molecule, as one of the important pathways regulating cell proliferation, is also involved in cell apoptosis (48). It has reported that the phosphorylation of BAD is inhibited by PI3K/Akt signaling molecules, resulting in cell apoptosis (49). Meanwhile, the activation of caspase-9 can be suppressed by inhibiting the phosphorylation of caspase-9 (50). In addition, the release of cytochrome C from the mitochondria into the cytoplasm is also inhibited by the PI3K/Akt signaling pathway (51). In our study, we found that cinobufagin downregulated the expression of PI3K, p-PI3K, AKT, and p-AKT, indicating that the PI3K/AKT signaling pathway is also involved in cinobufagin-induced A375 cell apoptosis.

However, some limitations of this study should be noted. A melanoma-xenografted model in nude mice should also be used in future studies to evaluate the anti-tumor effects of cinobufagin. In conclusion, our results indicated that cinobufagin can inhibit A375 cell proliferation. In addition, cinobufagin upregulated ATM and Chk2, and downregulated CDC25C, leading to cell cycle arrest at the G2/M phase. Meanwhile, cinobufagin induced A375 cell apoptosis through the mitochondria-mediated apoptotic signaling pathway and inhibition of the PI3K/AKT signaling pathway. These results lay a solid foundation for the development of cinobufagin as a potential drug for the treatment of cutaneous malignant melanoma.

## Data Availability

All datasets generated for this study are included in the manuscript and/or the supplementary files.

## Author Contributions

ZP, XZ, and PY designed the study, acquired the data, and wrote the manuscript. XC, PL, ML, and XL collected cell samples for Hoechst 33258 staining, cell cycle, and western blot analyses. ZL, FW, and KW interpreted and analyzed the data. DL and QZ revised and approved the final version of the manuscript. All authors read and approved the manuscript and agree to be accountable for all aspects of the research in ensuring that the accuracy or integrity of any part of the work are appropriately investigated and resolved.

### Conflict of Interest Statement

The authors declare that the research was conducted in the absence of any commercial or financial relationships that could be construed as a potential conflict of interest.
